# A call for discovery: Re‐envisioning The Cancer Genome Atlas as a blueprint for a TCGA2.0—The COVID‐19 Genome Atlas

**DOI:** 10.1002/ctd2.7

**Published:** 2021-10-18

**Authors:** Kristian M. Hargadon

**Affiliations:** ^1^ Hargadon Laboratory Department of Biology Hampden‐Sydney College Hampden‐Sydney Virginia USA

## Abstract

The COVID‐19 pandemic has impacted the health of millions and had a myriad of devastating consequences for global societies since its emergence in 2019. Noting parallels between the impact of COVID‐19 and cancer as diseases of global health significance, and as a way of building off the successes of The Cancer Genome Atlas (TCGA) as a comprehensive, multiomics approach to understand and combat cancer, this Call For Discovery provides a vision for creating a new TCGA2.0 (The COVID‐19 Genome Atlas) as a tool that will benefit researchers, clinicians, and patients alike as the scientific community works to better understand not only the various determinants of COVID‐19 disease outcome but also the most effective ways to manage and treat COVID‐19 disease complications.

## 1 A NEED FOR DISCOVERY

With the launch of *Clinical and Translational Discovery* as a journal that aims to be a leading forum for research efforts to guide the future of molecular and precision‐based medicine, we find ourselves at a time in history when the importance of medical discovery could not be more relevant to our global society. In the midst of a COVID‐19 pandemic that has already claimed at least 4.7 million lives since the first cases of disease were reported in the late fall of 2019, there is an urgent need to better understand the array of factors that ultimately dictate disease outcome, not only for the purpose of identifying still unknown risk factors for severe illness but also for the purpose of developing novel therapeutic strategies to interfere with disease progression. Though the scope of COVID‐19, encompassing everything from asymptomatic cases to death, has challenged much of society's collective perceptions of—and overall response to—the pandemic, the scientific community has never been more poised to capitalize on technological advances that will enable the very discovery necessary to understand the molecular determinants of such a diverse range of disease presentation. Particularly in this era of high throughput, omics‐based discovery, the ability to generate and analyze large datasets from clinical specimens offers an opportunity to answer several important questions surrounding COVID‐19. Why do some patients infected with SARS‐CoV‐2 remain asymptomatic while others require hospitalization and, in many cases, succumb to their illness? Which virus‐specific and host‐intrinsic factors are the most critical determinants of SARS‐CoV‐2 infection/replication and COVID‐19 disease progression? What accounts for the rapid resolution of disease in some patients versus the development of long‐haul COVID‐19 symptoms in others? While vaccination remains the gold standard in terms of COVID‐19 prevention, addressing these issues is also critical for minimizing disease impact in patients who ultimately decline the available vaccines, patients not yet eligible for vaccination, and patients for whom vaccination may not confer complete protection. Together with efforts to increase acceptance of the highly efficacious SARS‐CoV‐2 vaccines, discovering the answers to these and related questions is indeed what will guide us through, and ultimately lead us out of, the current pandemic.

Many insights into COVID‐19 pathology undoubtedly lie in understanding SARS‐CoV‐2‐specific virulence factors and indeed are emerging from studies evaluating the functions of specific viral gene products. Such studies have identified important roles for several structural and nonstructural SARS‐CoV‐2 proteins in processes associated with virus pathogenicity, including: preferential translation of viral, rather than host protein^1^, ^2^; molecular mimicry of host RNA as a means of evading immunity‐inducing innate RNA sensors^3^; and direct antagonism of the interferon antiviral defense system.^4^, ^5^ At the same time, others have documented critical elements of the host that also influence SARS‐CoV‐2 pathogenicity and COVID‐19 disease severity. Among those host‐intrinsic factors associated with severe disease are hyperinflammatory gene and protein expression signatures and altered immune cell profiles including T lymphopenia and a shifted distribution of innate immune cell populations.^6^, ^7^, ^8^, ^9^, ^10^ In keeping with the relevance of type I interferon immunity to the outcome of SARS‐CoV‐2 infection, recent studies in independent patient cohorts have also documented an enrichment of neutralizing type I interferon autoantibodies in patients who experience severe/critical COVID‐19 versus those who remain asymptomatic or experience only mild disease following SARS‐CoV‐2 infection.^11^, ^12^ Likewise, transcriptomic and protein‐based analyses of patient samples have revealed altered expression of interferon pathway genes as a correlate of severe COVID‐19 illness,^13^, ^14^ and genomic profiling studies have identified inborn errors in several interferon pathway genes in critically ill COVID‐19 patients.^15^, ^16^, ^17^ Importantly, in two of these studies the genomic defects in interferon pathway genes were found to be significantly enriched in critically ill patients as compared to those with asymptomatic/benign disease.^16^, ^17^ Of note, however, this latter observation could not be confirmed by an independent study,^18^ underscoring the need for more comprehensive analyses of larger datasets to: (1) validate the significance of this and other correlates of COVID‐19 disease severity in broad patient populations and (2) provide insight into the most relevant biomarkers and mechanisms of COVID‐19 disease progression.

## 2 A FOUNDATION FOR DISCOVERY

This inaugural issue of *Clinical and Translational Discovery* comes nearly two decades removed from completion of one of the greatest accomplishments of modern science, the Human Genome Project–a landmark undertaking that spanned 15 years and which ultimately sparked an ‐omics revolution in scientific discovery. The insight into human genetics and the technological advances spawned by this historic scientific achievement have significantly advanced our understanding of the cellular and molecular processes underlying human health and disease, and they have transformed the ways in which we approach scientific inquiry, prompting reverse translational research at an unprecedented scale that, in turn, continues to drive bench‐to‐bedside application of new discoveries.

Perhaps no clearer example of the Human Genome Project's impact on advancing our understanding of human disease is the foundation it set for subsequent development of The Cancer Genome Atlas (TCGA), an endeavor that molecularly characterized over 20,000 tumor and matched normal tissue samples across 33 distinct cancer types.^19^ Initiated in 2005 on the heels of the Human Genome Project and completed in less time than that needed to sequence the first human genome, this atlas of cancer includes not only genomic characterization of the most common tumor types but also transcriptomic, epigenomic, and proteomic data that continue to provide important insights into the development and progression of many cancers. These data have been used to identify novel cancer driver genes, assess the impact of dysregulated gene expression on cancer progression, define core cancer‐promoting signaling pathways whose activity is influenced by genomic and transcriptional alterations in diverse genes, reclassify once‐broadly‐categorized cancer types into molecular subtypes, determine specific mediators of tumor drug resistance and immune evasion, identify biomarkers predictive of disease progression, and inform the development and application of precision‐based targeted and immune therapies for cancer patients. Moreover, in addition to those discoveries unique to particular tumor types and even tumor subtypes, concomitant efforts to evaluate the collective TCGA dataset in the form of a Pan‐Cancer Atlas has also revealed common themes across cancer biology,^20^ highlighting shared features of diverse cancer types and identifying common molecular influences on the hallmark properties of cancer cells.^21^ Finally, perhaps equally important as the biological insights made possible by analysis of TCGA data is the model for future discovery set forth by the TCGA project itself as a collaborative, international, multidisciplinary effort to address a common scientific problem. It is in this vein that lessons learned from the TCGA program have the potential to significantly impact our response to other scientific challenges of global health concern, including the ongoing COVID‐19 pandemic.

## 3 A CALL FOR DISCOVERY

**FIGURE 1:**
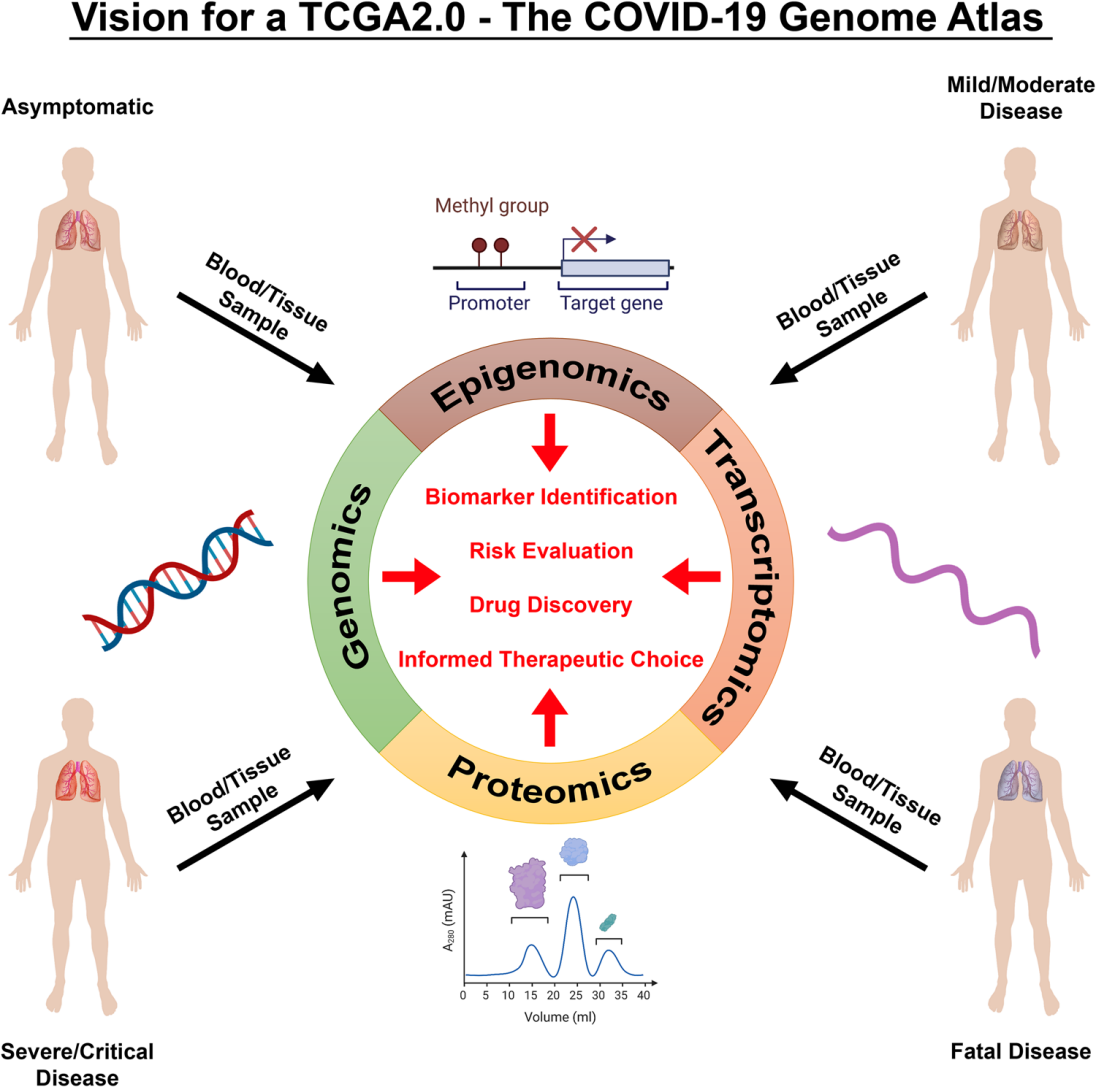
A vision for a TCGA2.0–The COVID‐19 Genome Atlas. A large‐scale, comprehensive multiomics approach to study COVID‐19 has the potential to transform our understanding and treatment of COVID‐19 in much the same ways the original TCGA has informed our understanding and clinical management of cancer. Genomic, epigenomic, transcriptomic, and proteomic analyses of blood and tissue samples derived from patients across the clinical spectrum of COVID‐19 disease presentation will identify biomarkers predictive of disease progression, aid clinicians and patients with risk evaluation, drive drug discovery and repurposing efforts, and improve therapeutic decision‐making. Portions of this figure were created with BioRender.com

Cancer and COVID‐19 are in many ways polar opposites in terms of their classification as human diseases. On the one hand, cancer is a disease of abnormal self cells that arise from genetic aberrations within; COVID‐19, on the other hand, is a disease of nonself, triggered by infection with a foreign pathogen. Cancer is a noncommunicable disease, while COVID‐19 is highly transmissible. Cancer typically develops over years, whereas COVID‐19 causes illness, and even death, within days to weeks of SARS‐CoV‐2 infection. Despite these distinct differences, however, there are also important parallels that exist between cancer and COVID‐19. While both are more likely to impact the elderly, they can also strike persons of any age. Both present not only health challenges but also social, emotional, and economic challenges that impact affected individuals, their friends and families, and even entire healthcare systems. Both are associated with highly variable clinical outcomes, a factor that complicates prognosis and that can result in unnecessary treatment in some cases and insufficient treatment in others. In light of these parallels, this Call for Discovery accentuates the need for the scientific community to leverage its collective efforts to combat COVID‐19 in much the same way it has cancer. Indeed, it offers a vision for how the original TCGA can now serve as a blueprint for creation of a new TCGA2.0–The COVID‐19 Genome Atlas (Figure 1), which has the potential to improve our understanding of, and response to, COVID‐19 in many ways:

1. Just as genomic surveillance of SARS‐CoV‐2 has been useful for tracking the emergence of new virus variants, multiomics‐based surveillance of blood and tissue samples from patients afflicted with COVID‐19 will provide insight into host factors that influence disease resolution versus progression.
2. A collaborative, large‐scale atlas of genomic, epigenomic, transcriptomic, and proteomic correlates of COVID‐19 disease outcomes will be useful not only for validating findings from smaller studies that have already been conducted but also for identifying the most common correlates of severe disease and long‐term complications of COVID‐19.
3. A comprehensive COVID‐19 atlas has the potential to reveal specific correlates of severe disease that may be unique to particular demographics, an outcome unlikely to emerge from analysis of smaller datasets.
4. A broad molecular characterization of host‐intrinsic determinants of risk for COVID‐19 complications is likely to reveal clinically silent biomarkers that will offer insight into disease progression in patients who are young and/or who do not present with underlying health conditions currently known to place them at high risk.
5. Collectively, these findings will suggest new therapeutic targets for the treatment of COVID‐19, driving drug discovery and repurposing efforts to improve disease management and patient outcome.

Just as cancer is in some ways an inevitable part of our being, nearly 2 years into this pandemic it is already becoming clear that COVID‐19 is likely to become a permanent part of our existence as well. Yet we continue to make great progress in the fight against cancer, and we will make great progress against COVID‐19 too. The Call for Discovery outlined herein is intended to galvanize efforts toward this very progress, with the hope that *Clinical and Translational Discovery* can play a significant role in sharing such important discoveries with the scientific community. It is this discovery that will enable physicians, epidemiologists, and patients alike to make more informed decisions about treatment, public health recommendations, and personal behaviors. It is this discovery that will yield new therapeutics that interfere with virus‐associated pathology and disease progression. And it is this discovery that will advance ongoing efforts to combat COVID‐19 and the ever‐evolving SARS‐CoV‐2 coronavirus, efforts that will ultimately bring this pandemic to its end and, in turn, lessen the individual and global impact of this devastating disease.

## CONFLICT OF INTEREST

The author declares no conflict of interest.

## Data Availability

Data sharing is not applicable to this article as no datasets were generated or analyzed for the purposes of this Editorial article.
